# Preparation and Characterization of Solid Dispersions Composed of Curcumin, Hydroxypropyl Cellulose and/or Sodium Dodecyl Sulfate by Grinding with Vibrational Ball Milling

**DOI:** 10.3390/ph13110383

**Published:** 2020-11-12

**Authors:** Nguyen Ngoc Sao Mai, Yuta Otsuka, Yayoi Kawano, Takehisa Hanawa

**Affiliations:** Department of Pharmaceutical Sciences, Faculty of Pharmaceutical Sciences, Tokyo University of Science, 2641 Yamazaki, Noda, Chiba 278-8510, Japan; apricotd2003@gmail.com (N.N.S.M.); y.otsuka@rs.tus.ac.jp (Y.O.)

**Keywords:** amorphous solid dispersion, solubility, stability, poorly water-soluble drug, vibrational ball milling, Fourier transform infrared spectroscopy, X-ray powder diffraction, differential scanning calorimetry, dissolution

## Abstract

Solubility is an important physicochemical property affecting drug bioavailability. One approach to improve drug solubility is using amorphous formulations, which can improve solubility by up to a 1000-fold. Herein, amorphous curcumin (CUR) and amorphous solid dispersions (SDs) consisting of CUR, hydroxypropyl cellulose (HPC) and/or sodium dodecyl sulfate (SDS) were developed using vibrational ball milling. The resulting ground mixtures (GMs) were characterized using powder X-ray diffractometry, Fourier transform infrared spectroscopy, differential scanning calorimetry and a dissolution test. The 60-min GM containing 90% HPC significantly increased the drug solubility. Presence of SDS in the GMs containing 90% HPC reduced the grinding duration from 60 min to 30 min in forming a ground SD that significantly increased the CUR dissolution rate. This amorphous state was stable for 30 days when stored at 40 °C/RH 75%.

## 1. Introduction

Solubility is one of the most important physicochemical properties affecting drug bioavailability. It is estimated that approximately 70% of the current new drug candidates have poor solubility [1]. One methodology that can enhance the solubility of poorly water-soluble drugs and their bioavailability is amorphization [2,3,4,5]. Indeed, the solubility of amorphous drugs can be up to a 1000-fold higher than that of their crystalline forms [6,7,8,9]. Amorphous substances can be classified into two categories: molecularly pure drugs and solid dispersions (SDs), where the active pharmaceutical ingredient (API) is dispersed within a carrier(s). Vo et al. have demonstrated that SDs can be categorized into four distinct subcategories [10]: (1) SDs that use a crystalline carrier, such as urea [11,12,13] or sugar [11]; (2) SDs that use a polymeric carrier, such as povidone [14], polyethylene glycol [15], hydroxypropyl cellulose [16] or cyclodextrin [17,18,19,20,21]; (3) SDs that use a carrier with surface activity, such as surfactant(s) or a mixture of polymers and surfactants [22]; and (4) controlled release SDs or SDs that use swellable/water insoluble polymers, such as Eudragit^®^ [23] and Carbopol^®^ [23].

In this study, we focused on curcumin (CUR) as a model of a poorly water-soluble API. CUR is a diarylheptanoid consisting of two aromatic rings joined by a seven-carbon chain. CUR exhibits various pharmacological properties, such as analgesic, anti-inflammatory, anti-oxidant, anti-malarial and insect repellant activities [24,25,26,27,28,29]. CUR has a low aqueous solubility (~28.9 ng/mL) and is dramatically metabolized through the first pass in the liver (maximum serum concentration at 1 h: 0.006 ± 0.005 µg/mL) [30]. Particularly, CUR has been used in accelerating the wound healing process. Kulac et al. described CUR as a wound dressing agent that protected wound tissue from microbial infection, reduced inflammation and stimulated cell proliferation to re-build damaged tissue [31]. Cianfruglia et al. reported that CUR at a high concentration (≥20 µM) reduced the cell viability of human dermal fibroblasts (HDFs) with regard to the control by 57% [32]. The protective effect on HDF from ultraviolet A (UVA) was recorded at 5 µM CUR pretreated for 2 h prior to 10 J/m^2^ UVA irradiation exposure [33].

Hydroxypropyl cellulose (HPC) is used in SD formation to improve drug solubility. Yamada et al. demonstrated that the co-ground mixtures (consisting of 3, 9-bis(*N*, *N*-dimethylcarbamoyloxy)-5*H*-benzofuro (3, 2-c)quinoline-6-one and HPC) increased the dissolution rate and maintained the high drug solubility by increasing the particles’ surface area and forming an amorphous state [34]. Sugimoto et al. reported that co-ground phenytoin with L-HPC using ultra-cryo milling exhibited a dissolution rate of approximately 90% over 60 min [35]. Sodium dodecyl sulfate (SDS) is an anionic surfactant widely used to improve drug wettability, solubility and dissolution rate of poorly soluble APIs. Madelung et al. reported that felodipine and griseofulvin solubility linearly increased as the amount of SDS increased [36]. A small addition of SDS resulted in a significant increase in the dissolution rate of fenofibrate samples obtained by salt-assisted milling [37]. For prolonged contact with human skin, the SDS concentrations should not exceed 1% to avoid irritation [38].

For decades, to enhance CUR solubility, numerous methodologies forming CUR SDs with polymers have been accessed. Li et al. ameliorated CUR solubility by forming spray-dried SDs of CUR in cellulose derivative matrices (hydroxypropyl methylcellulose acetate succinate-HPMC AS, carboxymethylcellulose acetate butyrate and cellulose acetate adipate propionate) [39]. Seo et al. reported that an SD of CUR and polyethylene glycol-15-hydroxystearate obtained by the conventional solvent evaporation method significantly increased the drug solubility at physiological pH [40]. Satomi et al. developed CUR SDs with HPC-SL and HPMC AS using wet milling and freeze-drying methods [41]. Wiegel et al. investigated CUR amorphous SDs with polyvinyl pyrrolidone, Eudragit E100, carboxymethyl cellulose acetate butyrate, HPMC and HPMC AS using spin coating, rotary evaporation and cryo-milling [42].

We considered vibrational ball milling, a dry milling method to develop ground and co-ground CUR. In addition, it is applicable not only in laboratory research but also in pilot- and industrial-scale studies [43]. During the grinding process, various parameters can influence the efficiency of grinding, such as the frequency of the vibration, type of grinding jar (volume and material), type of media (quantity, material and diameter), amount of powder filling, percentage of components and grinding duration. Design of experiment (DoE) is an approach that can determine cause and effect relationships by introducing all possible inputs/factors acting on the process and all output(s)/response(s) based on the aims and statistical tests performed. Then, the experimental resources are optimized while considering all the restrictions and limitations of the resources.

This study focuses on the effects of grinding duration, HPC and SDS in solubility enhancement of ground and co-ground CUR using vibrational ball milling. Here, the amorphization was observed in a molecularly pure drug, SDs that consist of a drug and a polymer and SDs that consist of a drug, polymer and surfactant. The interactions between CUR–CUR and CUR–HPC after the grinding was evaluated. The CUR formulations that significantly improved the drug dissolution rate were detected.

## 2. Results and Discussion

### 2.1. Power X-ray Diffraction (PXRD) Patterns

**Figure 1:**
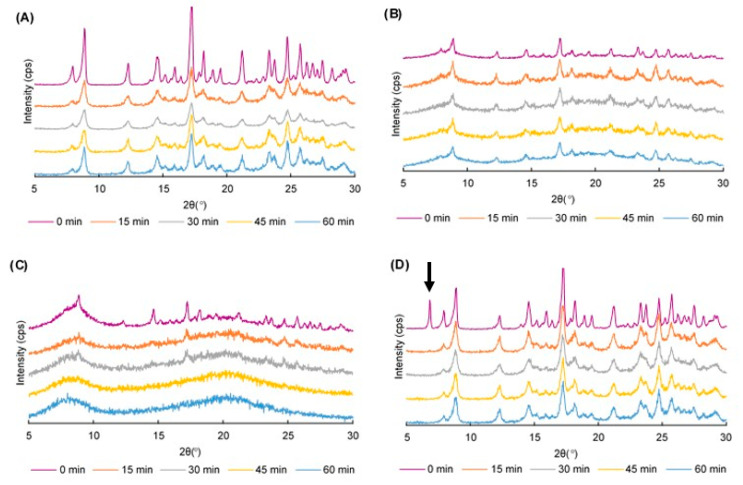
PXRD patterns of (**A**) ground curcumin (CUR) crystals; (**B**) ground mixtures (GMs) containing CUR and 50% hydroxypropyl cellulose (HPC); (**C**) GMs containing CUR and 75% HPC; and (**D**) GMs containing CUR and sodium dodecyl sulfate.

The PXRD patterns of 60 different samples were examined. The CUR crystals showed characteristic peaks at 2*θ* = 8.86, 12.28, 14.52 and 17.24°, which represented their crystalline nature (data are not shown). The PXRD pattern of HPC was observed as an amorphous form, whereas that of SDS showed a prominent peak at 6.8° (data are not shown). Figure 1 displays the PXRD patterns of ground CUR, ground mixtures (GMs) containing CUR and 50% HPC, GMs containing CUR and 75% HPC, and GMs containing CUR and SDS.

The CUR characteristic peaks could be observed irrespective of the grinding duration, but the peak intensity was decreased (Figure 1A). Colombo et al. [43] described that solid materials being processed in a mill received mechanical energy in a pulse form whenever they were entrapped between the media and mill wall. The transfer of mechanical energy through means of normal and shear stresses acting on the solid material surfaces created growth of a strain field in the solid bulk. The strain field-manifested atoms shift from the equilibrium stable positions at the lattice nodes or lattice collapse. Hence, the crystal transformation to an amorphous phase could be explained and the reduction in the peak intensity was greater as the grinding time was prolonged.

In binary ground mixtures (GMs) containing CUR and HPC (10, 25 and 50%), the characteristic peaks of CUR were visible but the reduction in the peak intensity clearly corresponded to the grinding duration (Figure 1B). An SD formation was observed in these GMs, but the CUR was not totally dispersed in HPC and reduced its crystallinity. In GMs containing CUR and 75% HPC, some prominent peaks were observed at 2*θ* = 17.24 and 24.76° for the 15-min and 30-min GMs. However, these peaks disappeared in the 45-min and 60-min GMs (Figure 1C). In the GMs containing CUR and 90% HPC, the characteristic peaks of CUR disappeared following 15 min of grinding (Figure S1F). The total transformation into an amorphous nature of the GMs showed that CUR was well dispersed within the matrix of the HPC as the amount of HPC was triple that of CUR.

In ternary physical mixtures (PMs) (containing CUR, HPC and SDS), regardless of the HPC amount, the prominent peak of SDS at 6.8° was observed (Figure 1D). However, this peak disappeared in all GMs (Figure S1D–L). Since the SDS amount in the mixtures was very small, its dispersion was easily attained. In other words, the presence of SDS did not clearly influence the crystallinity of the CUR in the GMs. As a result, the crystallinity transformation behavior of the ternary GMs was similar to that of the binary GMs.

### 2.2. Fourier Transform Infrared (FTIR) Spectroscopy

**Figure 2:**
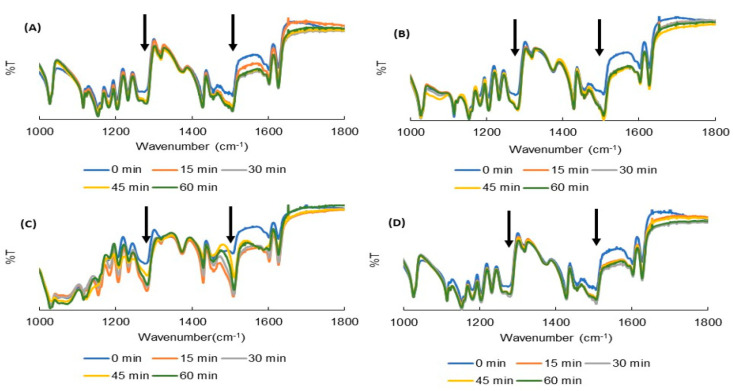
FTIR spectra of (**A**) ground curcumin (CUR) crystals; (**B**) ground mixtures (GMs) containing CUR and 50% hydroxypropyl cellulose (HPC); (**C**) GMs containing CUR and 75% HPC; and (**D**) GMs containing CUR and sodium dodecyl sulfate.

The FTIR spectra of the 60 samples were examined. The prominent peaks observed in CUR were as follows [44]: (1) 3502 cm^−1^ for the phenolic (-OH) vibrations; (2) 3017 cm^−1^ for the aromatic C–H stretching vibrations; (3) 1626 cm^−1^ for the conjugated alkene (C = C) stretching; (4) 1602 cm^−1^ for the stretching vibration of the benzene ring skeleton; (5) 1506 cm^−1^ for the mixed (C = O) and (C = C) vibrations; and (6) 1274 cm^−1^ for the methyl aryl ether (C–O) stretching vibrations (data are not shown). Figure 2 exhibits the FTIR spectra of ground CUR, ground mixtures (GMs) containing CUR and 50% HPC, GMs containing CUR and 75% HPC, and GMs containing CUR and SDS.

**Figure 3:**
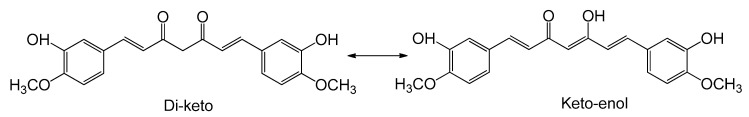
Keto-enol tautomer of curcumin.

In the ground CUR crystals, increases in the peaks’ intensity were observed at 1506 cm^−1^ and 1274 cm^−1^, corresponding to a carbonyl and ether group of CUR, respectively (Figure 2A). Peaks at 1274 cm^−1^ were also shifted to 1276, 1280, 1281 and 1281 cm^−1^, corresponding to data of 15-, 30-, 45- and 60-min GMs, respectively. Since CUR exists as keto-enol tautomer in the solid state (Figure 3) [45,46], it is considered that hydrogen bonding (H bond) was formed between CUR and CUR, with a hydrogen of enol (=C–OH) from one and an oxygen of the ether group (-OCH_3_) from another molecule. As the amount of HPC in the mixtures increased up to 50%, the variations in these peaks were also observed (Figure 2B). In addition to the CUR–CUR H bond, the H bonds between CUR and HPC (with the oxygen on the ether group of CUR and hydrogen on the -OH group of HPC, and the oxygen on the carbonyl group of CUR and hydrogen on the -OH group of HPC) co-existed.

Particularly, in GMs containing over 75% HPC (Figure 2C), new peaks at 1507 cm^−1^ and 1275 cm^−1^ were observed and became more intensive when the grinding duration increased. This suggests that the drug was thoroughly dispersed in the HPC and the only hydrogen bond between CUR and HPC was formed. In other words, the hydrogen bond between CUR and CUR did not exist. The SDS in both the ternary PMs and GMs did not influence the FTIR spectra of these GMs (Figure 2D and Figure S2D–L) because of its small amount and being over-imposed by the spectra of CUR and HPC.

### 2.3. Differential Scanning Calorimetry (DSC) Curves

**Figure 4:**
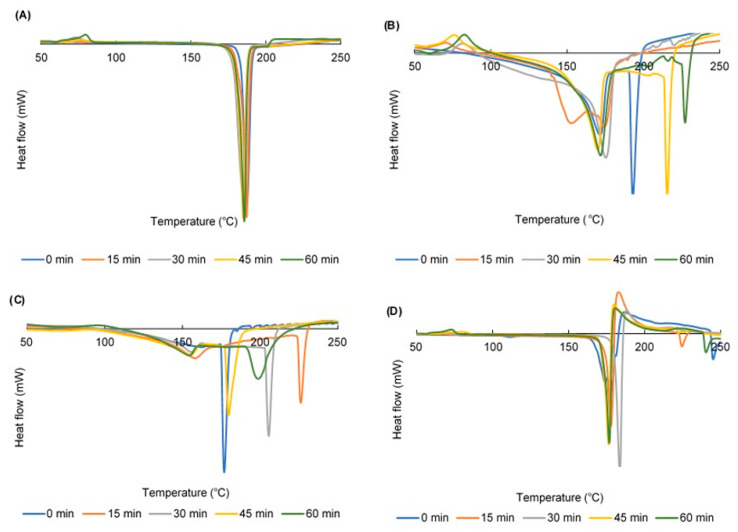
DSC curves of (**A**) ground curcumin (CUR) crystals; (**B**) ground mixtures (GMs) containing CUR and 50% hydroxypropyl cellulose (HPC); (**C**) GMs containing CUR and 75% HPC; and (**D**) GMs containing CUR and sodium dodecyl sulfate.

The DSC curve of the CUR crystals showed one endothermic at 187 °C, indicating their melting temperature, whereas the DSC graph of HPC exhibits one endothermic peak at 206 °C, corresponding to its decomposition temperature [47] (data are not shown). Figure 4 reveals the DSC curves of ground CUR, ground mixtures (GMs) containing CUR and 50% HPC, GMs containing CUR and 75% HPC, and GMs containing CUR and SDS.

DSC thermograms of the ground CUR crystals all showed melting peaks at approximately 190 °C (Figure 4A). These peaks were stable regardless of the grinding duration. However, exothermic peaks at approximately 80 °C were observed. It indicated the recrystallization of CUR since the grinding method transformed CUR from a crystalline to amorphous phase [43]. In addition, the longer the grinding duration, the higher the exothermic peaks heat flow was.

In the GMs, the DSC curves revealed both peaks of CUR and HPC, but they all were shifted to a lower temperature because the mixtures of CUR and HPC are considered as impure material [48]. As the percentage of HPC in the GMs increased, the melting peaks of CUR were broadened and more shifted to a lower temperature (Figure 4B,C). The recrystallization peaks could be observed in GMs containing up to 50% HPC.

In case of ternary GMs, the presence of SDS raised the right shoulders and un-stabilized the CUR melting peaks (Figure 4D). However, the recrystallization phenomenon of the ground CUR was similar to that of the binary GMs. In other words, the CUR recrystallization was prevented as the HPC amount in the GMs was over 75%.

### 2.4. Dissolution Profiles

**Figure 5:**
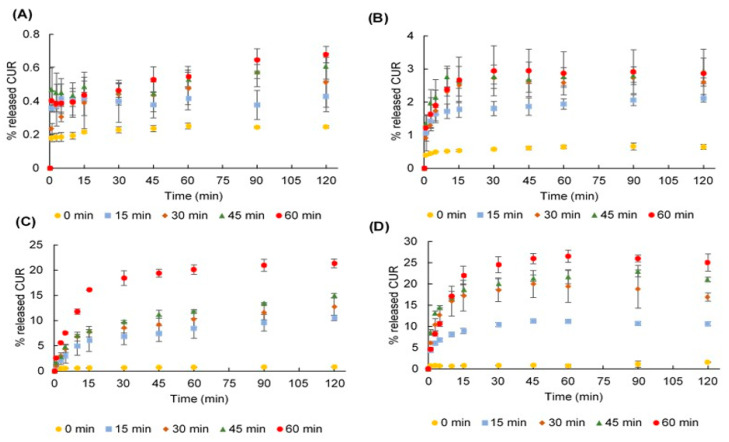
Dissolution profiles of (**A**) ground curcumin (CUR) crystals; (**B**) ground mixtures (GMs) containing CUR and 50% hydroxypropyl cellulose (HPC); (**C**) GMs containing CUR and 90% HPC; and (**D**) GMs containing CUR, 90% HPC and sodium dodecyl sulfate.

Dissolution profiles of the ground CUR crystals are shown in Figure 5A. As the grinding duration increased, the dissolution rate was enhanced, since CUR was partly transformed into an amorphous phase whose solubility was higher than the crystalline phase. Indeed, the percentage of released CUR over 120 min was ameliorated from 0.25 (ground for 0 min) to 0.68 (ground for 60 min).

Presence of HPC in PMs (0-min GMs) positively correlated with the dissolution rate of CUR (Figure 5B,C). Indeed, the released amounts of CUR over 120 min were 0.25, 0.55, 0.60, 0.64, 0.79 and 0.82%, corresponding to 0, 10, 25, 50, 75 and 90% HPC, respectively. In addition, the presence of SDS in ternary PMs could increase these numbers to 0.34, 0.65, 0.76, 0.95, 0.99 and 1.55%, correspondingly. The hydrophilicity of HPC and the surfactant characteristic of SDS can increase drug wettability and solubilization; thus, improve the CUR dissolution rate.

The dissolution rate of CUR in the GMs increased as the amount of HPC increased and as the grinding duration prolonged. The presence of SDS in ternary GMs also enhanced the CUR dissolution compared to the corresponding binary GMs. The greatest dissolution rate of CUR was observed in the 120-min GMs containing 90% HPC and 25% SDS (Figure 5D).

### 2.5. Fit Factors

Fit factors are used to evaluate the significant differences in dissolution rate of CUR between formulations and their corresponding PM. The dissolution data of the PMs were adopted as the reference during samples testing, which have the same amount of HPC and SDS. The difference factor (f_1_) and similarity factor (f_2_) values were calculated and are shown in Table 1. All samples had f_1_ values >15, indicating that the ground samples and presence of HPC and/or SDS in the samples led to a noticeable difference in CUR dissolution rate. In addition, an f_2_ value of <50 was observed in four GMs, namely, the 60-min binary GM containing 90% HPC (40.6) as well as the 30-min, 45-min and 60-min ternary GMs containing 90% HPC (corresponding to 40.4, 37.5 and 34.9, respectively). There were significant differences in the dissolution rate of these GMs. Therefore, the presence of SDS helped reduce the grinding duration from 60 min to 30 min in order to develop a ground SD, which significantly enhanced the drug solubility.

### 2.6. Dissolution Efficiency (DE)

DE (%) is a parameter reflecting the area under the dissolution curves. The DEs of the 60 samples were calculated and are shown in Table S1; the values were in the ranged between 0.23 and 23.97; the smallest value corresponded to the DE of CUR crystals, and the highest value corresponded to the DE of 60-min GM containing 90% HPC and SDS. Hence, the DE value of the later formulation increased 104 times as compared to the DE value of the CUR crystals.

In addition, because the DEs are comparative parameters to evaluate the differences among formulations, they were submitted into DoE as output data whereas the inputs were A-HPC (amount of HPC, %), B-grinding time (min) and C-presence of SDS. Following this, an analysis of variation was accessed to estimate the significance of the DoE model.

### 2.7. Analysis of DoE

**Figure 6:**
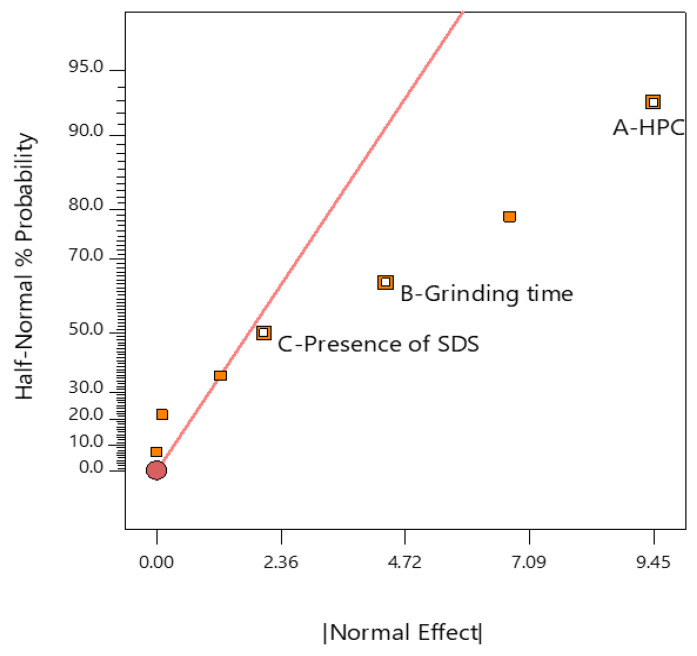
Half-normal plot of analysis. HPC, Hydroxypropyl cellulose; SDS, Sodium dodecyl sulfate.

The half-normal plot of effects helps to observe what factor is significant. The vertical (y) axis displays the cumulative probability of getting a result and the horizontal (x) axis reveals the absolute value of the effect [49]. Both data of the y and x axes were calculated by the DoE software. In the half-normal plot (Figure 6), A—HPC, B—grinding time and C—presence of SDS are shown as small squares on the right-hand side of the red line, referring to their positive effects on the response. In addition, the distances from these three factors to the red line were in order of A—HPC, B—grinding time and C—presence of SDS. This indicates that the influence of A–HPC on the DE was greater than that of B—grinding time and C—presence of SDS.

A model F-value of 13.77 implies model significance; there was only a 0.01% chance that an F-value this large could occur due to noise (Table 2). Because *p*-values > 0.05 indicate that the model terms are significant, A—HPC, B—grinding time and C—presence of SDS were significant model terms whose *p*-values were <0.0001, 0.0002 and 0.0465, respectively. In other words, A—HPC, B—grinding time and C—presence of SDS factors significantly influenced the DE. The smaller the *p*-value is, the greater is the influence of the factor on DE. This fact illustrated the mentioned analysis observed from the half-normal plot.

**Figure 7:**
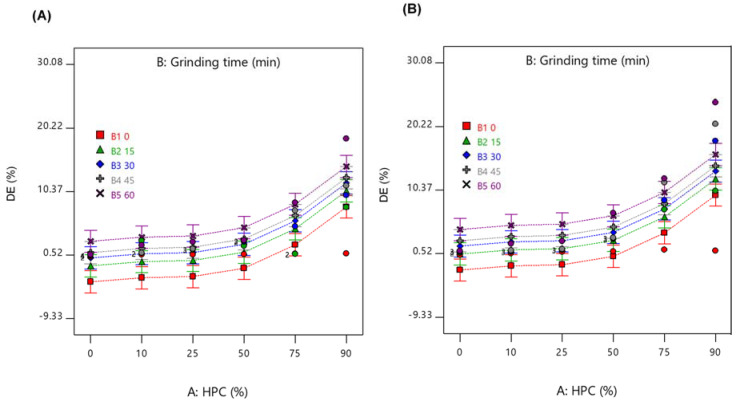
Interactions among factors of (**A**) Ground mixtures (GMs) without sodium dodecyl sulfate (SDS), and (**B**) GMs with SDS. B1: 0, 0-min grinding; B2: 15, 15-min grinding; B3: 30, 30-min grinding; B4: 45, 45-min grinding; and B5: 60, 60-min grinding.

The interactions of the three factors are shown in Figure 7. Samples ground for a longer duration expressed higher DE while samples containing a larger amount of HPC showed better DE. Moreover, the presence of SDS in the GMs significantly increased DE compared to GMs in the absence of SDS (*p*-value < 0.05).

### 2.8. Stability

**Figure 8:**
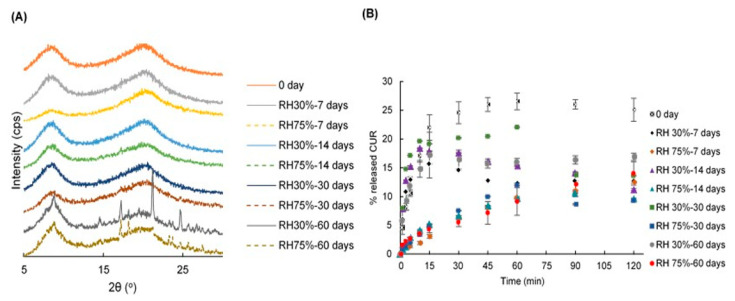
(**A**) PXRD patterns and (**B**) dissolution profiles of the 60-min ground mixtures containing HPC 90% and SDS after 0, 7, 14, 30 and 60-day storage at 40 °C/RH 30% and 40 °C/RH 75%.

The PXRD patterns and dissolution profiles of the 60-min GMs containing 90% HPC and SDS over 7, 14, 30 and 60 days at 40 °C/relative humidity (RH) 30% and 40 °C/RH 75% are shown in Figure 8. The halo patterns of the samples were stable up to 30 days in the stored conditions (Figure 8A). However, the CUR characteristic peaks appeared in samples after 60 days of storing. HPC may have protected the drug from crystallization for up to 30 days in the stored conditions.

After 7 days of storing, the dissolution rate of CUR was decreased (Figure 8B). In the condition of 30% RH, the CUR was continuously released over the initial period, ranging from 0 to 15 min and gradually decreased over the remaining time (from 30 to 120 min). However, samples stored in 75% RH released the drug very slowly during the 0–15 min initial duration followed by a gradual increase. Since the HPC can easily absorb water from the atmosphere, the samples become lumpy and slowly decomposed within 15 min. After 14 days of storing, the dissolution rate of CUR in 30% RH was higher than that in 75% RH. During the initial 15 min, CUR was gradually released, then decreased over the remaining time (from 30 to 120 min). The samples stored at RH 75% released CUR continuously over 120 min. However, after 30 days of storing, the samples in RH 30% progressively released CUR over 15 min, fluctuated during the period of 15 and 60 min and then decreased over the remaining time. Whereas, the samples in 75% RH released drug continuously over 60 min then decreased over the remaining time. In addition, after 60 days of storing, the samples in both 30% RH and 75% RH released CUR gradually over 120 min. Nevertheless, the sample dissolution rate in 75% RH was lower than that in 30% RH.

Concerning the restraints of this study, we used the DoE as a statistical equipment in analyzing the data and did not confer to its capacity of design experiments. Indeed, the accessed full factorial design of the DoE required 60 runs of the experiment. Instead, a fractional factorial design should be used, and the number of samples could be reduced to at least twice the number of runs [49]. This could reduce resources, but error effects and a power loss due to the reduction in runs are inevitable consequences [49].

## 3. Materials and Methods

Curcumin (CUR) was purchased from Tokyo Chemical Industry Co. Ltd. (Tokyo, Japan, >97%). HPC-L (molecular weight 140,000 Da) (from now on referred to as HPC) was provided by Nippon Soda Co. Ltd. (Tokyo, Japan). SDS and MeOH were purchased from Wako Pure Chemical Industries Co. Ltd. (Osaka, Japan) and Kanto Chemical Co, Inc (Tokyo, Japan), respectively. All substances were of analytical grade and no purification was carried out throughout the study.

### 3.1. Design of Experiments (DoE)

For the DoE study, 6 levels of HPC (0, 10, 25, 50, 75 and 90%), 5 different grinding times (0, 15, 30, 45 and 60 min) and 2 levels of SDS (without—0 and with—1) were selected as the inputs (Table 2). The SDS amount was 1% of the total amount of CUR and HPC. Design-Expert^®^ software version 11 and a full factorial design was utilized. These variables were set as categorical factors. The experimental design space consisted of 60 experiments (Table 1). All the DoE runs were performed randomly, using similar process conditions. The output data was the dissolution efficiency (DE), with the goal being to achieve the highest DE.

### 3.2. Preparation of Ground Mixtures (GMs)

The definite weight ratios of CUR, HPC and SDS were added to a 30 mL glass tube and mixed using a vortex mixer for 60 s to obtain the physical mixtures (PMs). A total of 300 mg of each PM was transferred to a 5 mL stainless-steel jar that would fit the MM400 mixer mill (Retsch, Haan, Germany), and that contained a ball (stainless steel, Φ7 mm). The jar was immersed in liquid nitrogen for 5 min and then the material was ground in the MM400 for 15 min at 30 Hz. The complete immersion for 5 min in liquid nitrogen and grinding process was repeated 1, 2, 3 and 4 times corresponding to 15, 30, 45 and 60 min of grinding duration, respectively. Following this, the co-GMs were sieved through a 36-mesh sieve and stored in a desiccator until further evaluation.

### 3.3. Quantification of CUR

CUR content was analyzed using UV-visible spectroscopy at 432 nm (Shimadzu 1800 UV-visible spectrophotometer, Shimadzu Co. Ltd., Kyoto, Japan). The analytical method was validated for specificity, linearity, accuracy and precision [50]. The standard CUR solution used for quantification was prepared in 50% (*v*/*v*) MeOH in distilled water (DW).

### 3.4. PXRD

The PXRD patterns were recorded using an PXRD (RINT 2000, Rigaku Co. Ltd., Tokyo, Japan) equipped with Ni-filtered CuKα radiation and operated at 40 kV and 40 mA. The diffractograms were recorded in the 2θ range from 5° to 30° at a scan rate of 2°/min.

### 3.5. FTIR

The attenuated total reflection method was used and FTIR spectra were recorded on a FTIR spectrometer Frontier(T)-UATR (DiKRS5) (Perkin-Elmer Inc., Shelton, CT, USA). The scanning range was 4000–500 cm^−1^ with 16 times the accumulation count, a 1 mm sample thickness and a resolution set at 1 cm^−1^. The obtained spectra were normalized using the SNV method [51].

### 3.6. DSC

DSC measurements were obtained using a DSC-60 Plus Differential Scanning Calorimeter (Shimadzu Co. Ltd., Kyoto, Japan) connected to a TA-60WS thermal analyzer (Shimadzu Co. Ltd., Kyoto, Japan) and an FC-60A flow controller (Shimadzu Co. Ltd., Kyoto, Japan). Approximately 3–5 mg of samples was weighed in aluminum pans and were sealed. An empty pan was used as the reference. The samples were scanned at 10 °C/min from 25 °C to 250 °C under N_2_ gas with a flow rate of 50 mL/min. The obtained spectra were normalized by the SNV method.

### 3.7. Dissolution Studies

The dissolution profiles of CUR in the GMs were evaluated using the dissolution apparatus as specified in the 17th Japanese Pharmacopoeia. GMs containing 5 mg of CUR were accurately weighed and placed in a vessel containing 500 mL of distilled water (DW). The temperature was controlled at 37 ± 0.5 °C and the rotating paddle speed was set at 100 rpm throughout this study. At defined time intervals (1, 3, 5, 10, 15, 30, 45, 60, 90 and 120 min), 5 mL aliquots were withdrawn, and an equal volume of fresh DW was added. The sample solutions were filtered through a 0.45-µm membrane filter and assayed for CUR content using UV-visible spectroscopy at 432 nm as described in the section “*Quantification of CUR*”. The release profiles of CUR were evaluated for the comparison. Each sample was carried out in triplicate.

### 3.8. Fit Factors

Moore and Flanner proposed procedures including a different factor and similar factor to compare the dissolution profiles in a pairwise fashion [52]. The difference factor (f_1_) measures the percent error between two curves over all time points (Equation (1)). The similarity factor (f_2_) is a logarithmic transformation of the sum-squared error of differences between the test T_t_ and reference samples *R_t_* over all time points (Equation (2)).

(1) $$f_{1}=\frac{\sum_{t=1}^{n}\left|R_{t}-T_{t}\right|}{\sum_{t=1}^{n}R_{t}}$$

(2) $$f_{2}=50\times log\left[\frac{100}{\sqrt{\left(1+\frac{1}{n}\sum {\left(R_{t}-T_{t}\right)}^{2}\right)}}\right]$$

where *n*: number of time points; *R_t_*: the mean dissolution value (%) for the reference product at time *t*; and *T_t_*: the mean dissolution value (%) for the test product at the same time *t*.

The different factor f_1_ is 0 when the test and reference profiles are identical and increase proportionally with the dissimilarity between two dissolution profiles. The similar factor f_2_ fits the result between 0 and 100. It is 100 when the test and the reference profiles are identical and tends to 0 as the dissimilarity increases. In general, f_1_ values lower than 15 (0–15) and f_2_ values higher than 50 (50–100) show the similarity of the dissolution profiles [53].

In this study, to identify the formulations and conditions of the grinding method that can significantly enhance the kinetic solubility of drug, the f_1_ and f_2_ should represent the dissimilarity of the dissolution profiles. In other words, an f_1_ value higher than 15 and f_2_ values lower than 50 are noticed.

### 3.9. DE

Khan and Rhodes suggested DE as a suitable parameter for the evaluation of in vitro dissolution [54]. The term DE for a pharmaceutical dosage form is defined as the area under the dissolution curve up to a certain time *t*, expressed as a percentage of the area of the rectangle described by 100% dissolution at the same time. It can be calculated using the following Equation (3):

(3) $$\mathrm{DE}\left(\%\right)=\frac{\int_{0}^{t}y\times dt}{y_{100}\times t}\times 100$$

where *y* is the drug percent dissolved at time *t*.

Here, we considered both the shape of the dissolution curve and the rate of dissolution, rather than the latter parameter only. The DE summarizes the drug release data into a single figure that is ready to make a comparison between a large number of formulations [54]. The resulting DE values were submitted to the DoE in order to detect the existence of significant differences between the formulations.

### 3.10. Stability Study

To evaluate stability, the 60-min GM containing 90% HPC and SDS was stored for 60 days at 40 °C/30% RH and 40 °C/75% RH. The PXRD and dissolution profiles were examined at predetermined time intervals over this period (0, 7, 14, 30 and 60 days).

## 4. Conclusions

In this study, amorphization of CUR and CUR SDs consisting of CUR, HPC and/or SDS were developed by vibrational ball milling. The resulting ground samples were characterized using PXRD, FTIR, DSC and a dissolution test. The 60-min GM containing 90% HPC significantly increased the CUR solubility. Presence of SDS in GMs containing 90% HPC reduced the grinding duration from 60 min to 30 min in forming a ground SD that significantly increased the drug dissolution rate. This amorphous state was stable for 30 days when stored at 40 °C/RH 75%.

## Figures and Tables

**Table 1:** Fit factors.

**Without SDS**												
**Grinding Time (min)**	**HPC 0%**		**HPC 10%**		**HPC 25%**		**HPC 50%**		**HPC 75%**		**HPC 90%**	
	**f_1_**	**f_2_**	**f_1_**	**f_2_**	**f_1_**	**f_2_**	**f_1_**	**f_2_**	**f_1_**	**f_2_**	**f_1_**	**f_2_**
15 min	54.8	99.6	48.4	99.1	70.8	93.7	77.2	88.5	81.7	82.3	91.9	59.8
30 min	56.6	99.5	58.3	98.2	66.0	92.3	82.0	83.3	90.3	68.2	93.5	54.9
45 min	63.5	99.2	59.8	97.8	72.9	92.6	84.0	81.3	93.1	59.9	94.3	51.8
60 min	63.2	99.2	81.6	87.7	84.8	82.2	83.7	81.5	93.8	57.0	96.6	40.6
**With SDS**												
**Grinding time (min)**	**HPC 0%**		**HPC 10%**		**HPC 25%**		**HPC 50%**		**HPC 75%**		**HPC 90%**	
	**f_1_**	**f_2_**	**f_1_**	**f_2_**	**f_1_**	**f_2_**	**f_1_**	**f_2_**	**f_1_**	**f_2_**	**f_1_**	**f_2_**
15 min	59.1	98.8	54.8	98.3	47.8	97.6	78.2	81.8	66.2	65.9	92.0	53.5
30 min	67.4	97.7	55.1	98.2	54.9	96.4	78.9	80.9	71.1	60.8	95.5	40.4
45 min	73.2	96.8	63.4	96.8	54.5	96.5	79.0	80.8	75.7	54.7	96.1	37.5
60 min	74.0	96.5	82.5	86.8	78.1	84.5	90.8	61.5	76.4	53.4	96.3	34.9

SDS, Sodium dodecyl sulfate; HPC, Hydroxypropyl cellulose; f_1_, Difference factor; f_2_, Similarity factor.

**Table 2:** Result of the analysis of variation.

Source	Sum of Squares	df	Mean Square	F-Value	*p*-Value	
Model	1314.82	10	131.48	13.77	<0.0001	significant
A—HPC	1008.40	5	201.68	21.12	<0.0001	
B—Grinding time	266.59	4	66.65	6.98	0.0002	
C—Presence of SDS	39.84	1	39.84	4.17	0.0465	

df, Degrees of freedom; HPC, Hydroxypropyl cellulose; SDS, Sodium dodecyl sulfate.

## Supplementary Materials

The following are available online at https://www.mdpi.com/1424-8247/13/11/383/s1, Figure S1. PXRD patterns of 60 samples, Figure S2. FTIR spectra of 60 samples, Figure S3. DSC curves of 60 samples, Figure S4. Dissolution behaviors of 60 samples, Table S1. Inputs and output data of design of experiment.
