# Supplementary material for: Preparation and Characterization of Solid Dispersions Composed of Curcumin, Hydroxypropyl Cellulose and/or Sodium Dodecyl Sulfate by Grinding with Vibrational Ball Milling
Source: Pharmaceuticals (Basel). 2020 Nov 12;13(11):383. doi: 10.3390/ph13110383 (PMC7696471; doi:10.3390/ph13110383)
Supplement: Supplementary file 1 [file pharmaceuticals-13-00383-s001.pdf]

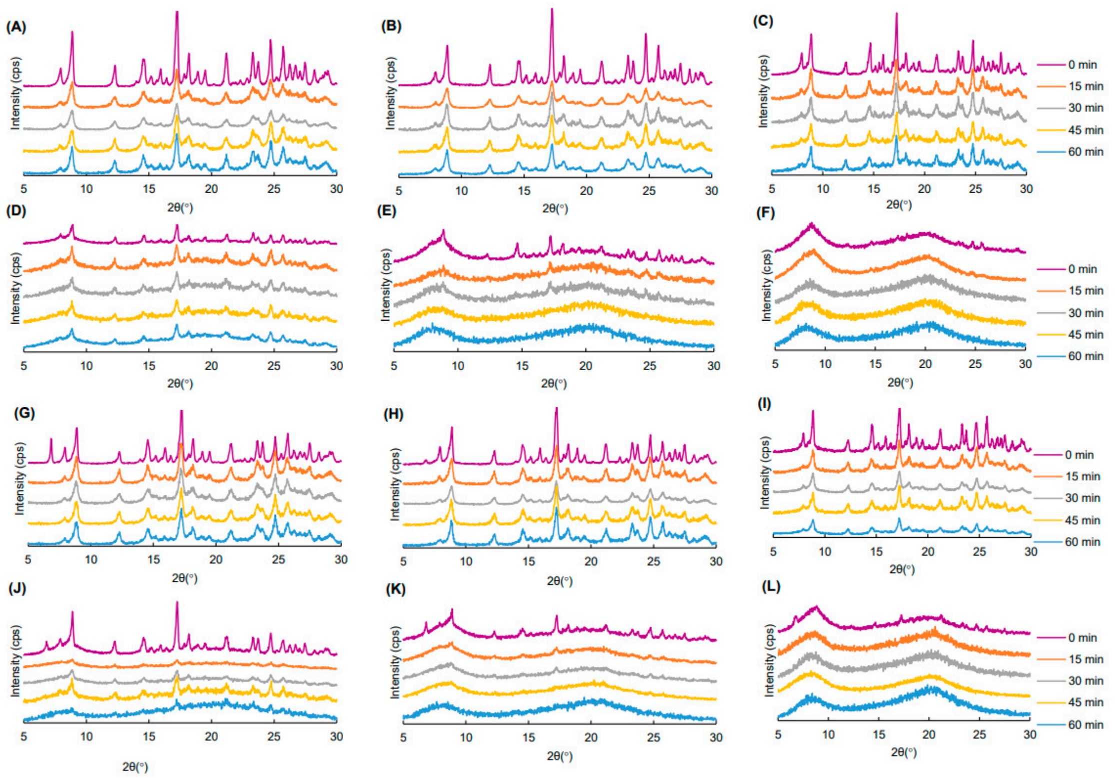

Figure S1. PXRD patterns of (A) ground curcumin (CUR) crystals; (B) ground mixtures (GMs) containing CUR and 10% hydroxypropyl cellulose (HPC); (C) GMs containing CUR and 25% HPC, and SDS; (D) GMs containing CUR and 50% HPC; (E) GMs containing CUR and 75% HPC; (F) GMs containing CUR and 90% HPC; (G) GMs containing CUR and sodium dodecyl sulfate (SDS), (H) GMs containing CUR, 10% HPC, and SDS; (I) GMs containing CUR, 25% HPC, and SDS; (J) GMs containing CUR, 50% HPC, and SDS; (K) GMs containing CUR, 75% HPC, and SDs; (L) GMs containing CUR, 90% HPC, and SDS.

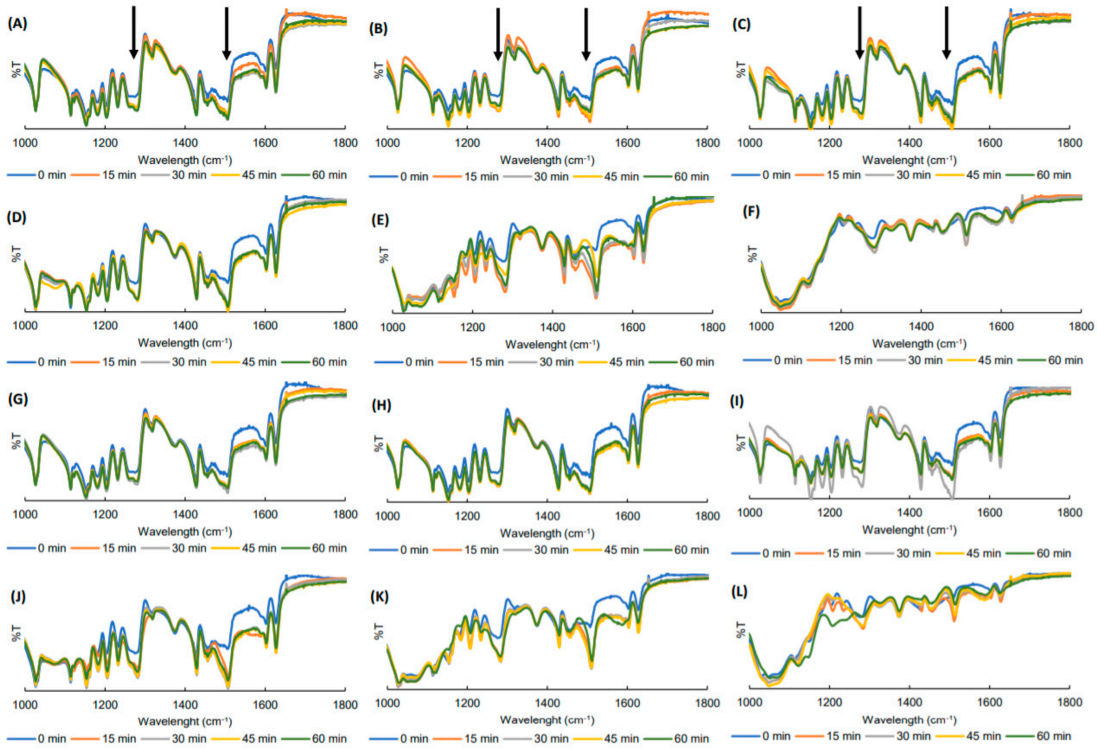

Figure S2. FTIR spectra of (A) ground curcumin (CUR) crystals; (B) ground mixtures (GMs) containing CUR and 10% hydroxypropyl cellulose (HPC); (C) GMs containing CUR and 25% HPC, and SDS; (D) GMs containing CUR and 50% HPC; (E) GMs containing CUR and 75% HPC; (F) GMs containing CUR and 90% HPC; (G) GMs containing CUR and sodium dodecyl sulfate (SDS), (H) GMs containing CUR, 10% HPC, and SDS; (I) GMs containing CUR, 25% HPC, and SDS; (J) GMs containing CUR, 50% HPC, and SDS; (K) GMs containing CUR, 75% HPC, and SDs; (L) GMs containing CUR, 90% HPC, and SDS.

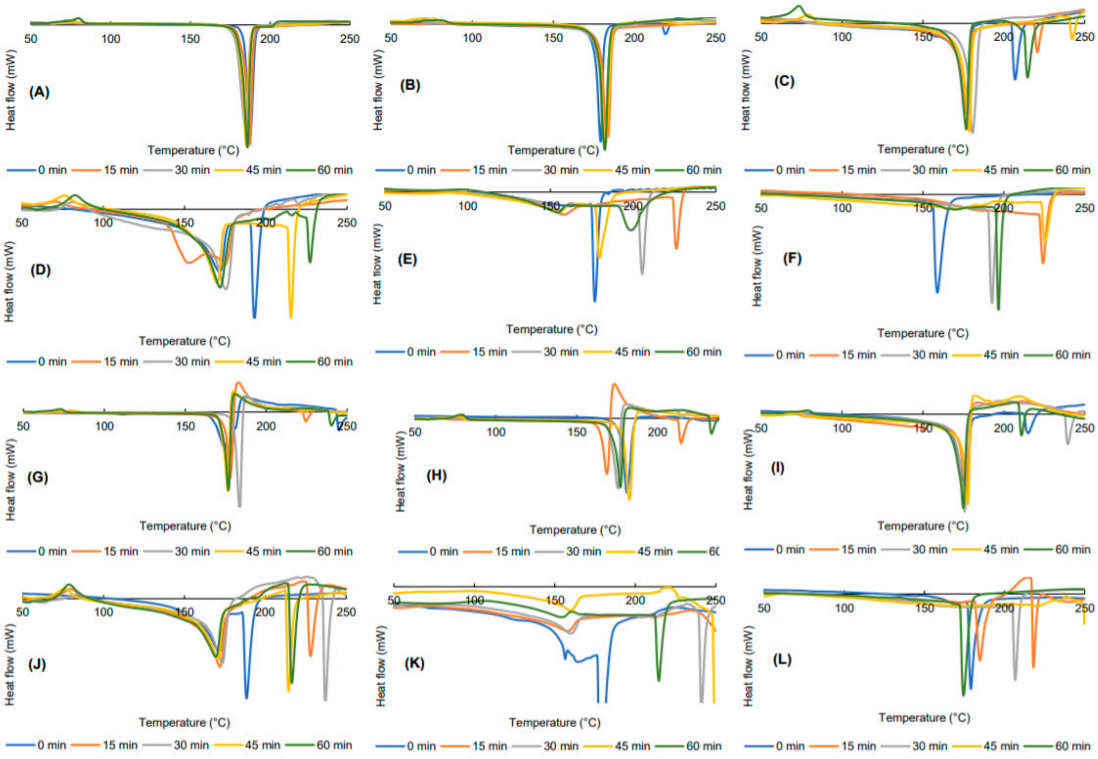

Figure S3. DSC curves of (A) ground curcumin (CUR) crystals; (B) ground mixtures (GMs) containing CUR and 10% hydroxypropyl cellulose (HPC); (C) GMs containing CUR and 25% HPC, and SDS; (D) GMs containing CUR and 50% HPC; (E) GMs containing CUR and 75% HPC; (F) GMs containing CUR and 90% HPC; (G) GMs containing CUR and sodium dodecyl sulfate (SDS), (H) GMs containing CUR, 10% HPC, and SDS; (I) GMs containing CUR, 25% HPC, and SDS; (J) GMs containing CUR, 50% HPC, and SDS; (K) GMs containing CUR, 75% HPC, and SDs; (L) GMs containing CUR, 90% HPC, and SDS.

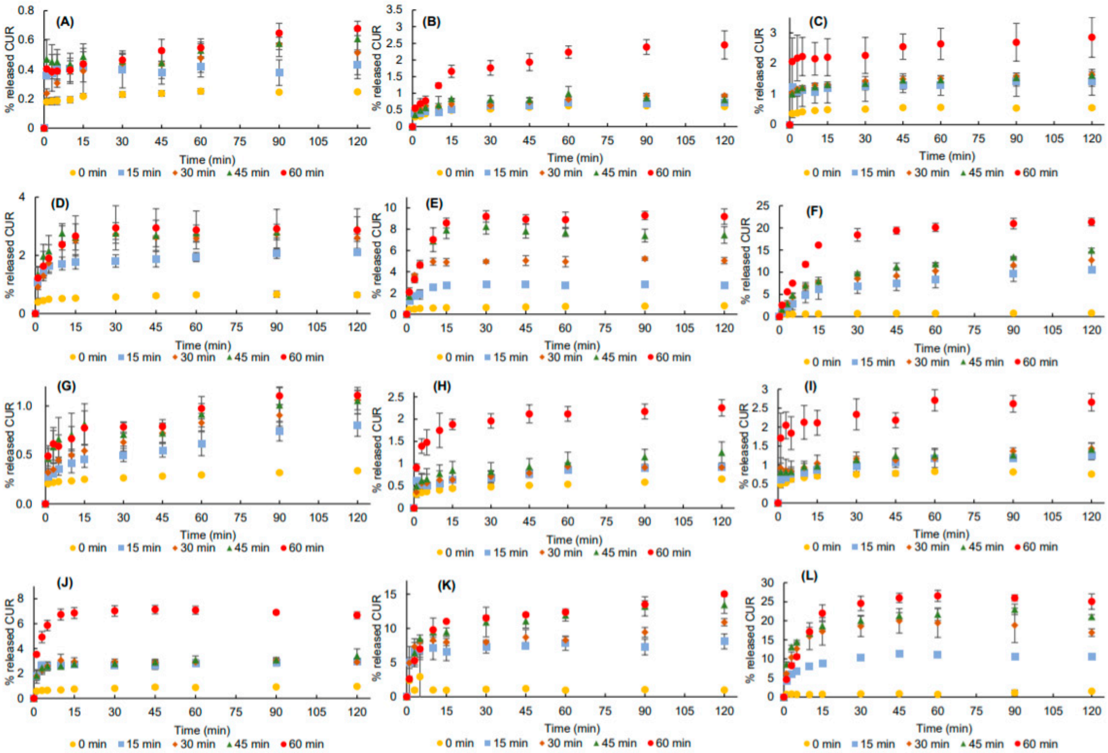

Figure S4. Dissolution profiles of (A) ground curcumin (CUR) crystals; (B) ground mixtures (GMs) containing CUR and 10% hydroxypropyl cellulose (HPC); (C) GMs containing CUR and 25% HPC, and SDS; (D) GMs containing CUR and 50% HPC; (E) GMs containing CUR and 75% HPC; (F) GMs containing CUR and 90% HPC; (G) GMs containing CUR and sodium dodecyl sulfate (SDS), (H) GMs containing CUR, 10% HPC, and SDS; (I) GMs containing CUR, 25% HPC, and SDS; (J) GMs containing CUR, 50% HPC, and SDS; (K) GMs containing CUR, 75% HPC, and SDs; (L) GMs containing CUR, 90% HPC, and SDS.

Table S1. Inputs and output data of design of experiment

	Factor 1	Factor 2	Factor 3	Response		Factor 1	Factor 2	Factor 3	Response
Run	HPC (%)	Grinding time (min)	Presence of SDS	DE (%)	Run	HPC (%)	Grinding time (min)	Presence of SDS	DE (%)
1	10	30	0	0.78	31	25	30	0	1.47
2	0	15	0	0.40	32	25	45	1	1.20
3	75	60	1	12.16	33	90	60	1	23.97
4	10	15	0	2.69	34	0	30	0	0.47

5	50	45	0	2.72	35	75	15	0	0.64
6	0	45	0	0.52	36	10	60	0	2.02
7	0	60	0	0.55	37	50	60	0	2.78
8	90	15	1	10.24	38	90	15	0	7.92
9	10	45	1	1.00	39	0	45	1	0.87
10	75	45	0	7.39	40	10	30	1	0.81
11	90	60	0	18.59	41	25	30	1	1.20
12	10	60	1	2.04	42	25	60	1	2.43
13	75	60	0	8.62	43	50	15	1	2.77
14	25	45	0	1.44	44	50	30	1	2.90
15	50	30	0	2.55	45	75	15	1	7.35
16	90	45	0	11.25	46	75	30	1	8.79
17	75	30	0	4.95	47	75	45	1	11.54
18	90	30	0	9.76	48	90	45	1	20.64
19	10	45	0	0.83	49	0	0	0	0.23
20	50	45	1	2.96	50	10	0	0	0.53
21	90	30	1	17.99	51	25	0	0	0.54
22	0	15	1	0.61	52	50	0	0	0.61
23	25	60	0	2.53	53	75	0	0	0.69
24	25	15	0	1.31	54	90	0	0	0.72
25	0	30	1	0.77	55	0	0	1	0.29
26	0	60	1	0.92	56	10	0	1	0.53
27	10	15	1	0.79	57	25	0	1	0.77
28	50	15	0	1.91	58	50	0	1	0.85
29	25	15	1	1.08	59	75	0	1	1.10
30	50	60	1	6.80	60	90	0	1	0.95

HPC, Hydroxypropyl cellulose; SDS, Sodium dodecyl sulfate; DE, Dissolution efficiency
